# Putting performance bias to the front-lower side of the hole on steep slopes: Differences in strategies and movements between professional and amateur golfers

**DOI:** 10.1371/journal.pone.0314820

**Published:** 2024-12-31

**Authors:** Yumiko Hasegawa, Ayako Okada, Keisuke Fujii

**Affiliations:** 1 Faculty of Humanities and Social Sciences, Iwate University, Iwate, Japan; 2 Japan Ladies Professional Golfers’ Association, Tokyo, Japan; 3 Graduate School of Informatics, Nagoya University, Nagoya, Aichi, Japan; 4 RIKEN Center for Advanced Intelligence Project, Suita, Osaka, Japan; 5 PRESTO, Japan Science and Technology Agency, Kawaguchi, Saitama, Japan; Shahid Chamran University of Ahvaz, IRAN, ISLAMIC REPUBLIC OF

## Abstract

When playing on an sloped golf course, the ball often misses the hole to the front-lower side, which is also referred to as the “amateur side.” This can be attributed to the difficulty in perceiving the green slope and choosing the appropriate launch direction and ball speed, especially for amateurs. The study had three main objectives. The first was to examine whether a front-down bias toward the hole could be experimentally observed in golfers’ putting. Second, to measure golfers’ aims and movements to determine why golfers, especially amateurs, putt poorly on steep slopes. Third, to investigate using a miniature platform how golfers recognized the steepness of the slopes and the relationship between their aim and their slope recognition. Twelve professionals and twelve intermediate amateurs were asked to perform in the 1- and 3-degree conditions (left-to-right line) at a distance of 3 m from the hole. Participants wore shutter goggles to inhibit visual feedback of outcomes. The aim, address, ball launch direction (analyzed as angles), and kinematics of the putter head and ball were measured. The results of this study experimentally confirmed the amateur side and indicated that the aim, address, and launch directions of the amateurs were smaller than those of the professionals. As for reproducing the slope task, we found that the amateurs underestimated the steepness on both conditions when compared to professionals. Additionally, golfers’ aim and slope recognition were weakly correlated. These suggest that amateurs, compared to professionals, were unable to choose the optimal speed of the putter head and the launch direction of the ball in relation to the slope. Furthermore, amateurs’ recognition was worse than that of professionals, which may affect their aim.

## Introduction

Skillful movement in sports requires integrating the physical characteristics of the environment (e.g., object placement, distance, gradient, etc.) with an action strategy that responds well to those characteristics. Golf putting requires golfers accurately to read how the putt will break on undulating greens and based on that information, apply the required force in the target direction. When putting on inclines, the putt often misses the hole to the front-lower side, which is sometimes referred to as the “amateur side.” Environmental perception and aim are inseparable in golf. For example, when the performer’s heels are high side toward the target, the ball breaks to the right, thus the performers must hit the ball to the left according to the amount of estimated break. Further, the ball’s roll is mostly determined by the launch angle and the force on the putter head at impact [1]. In other words, when putting on inclines, if performers hit the ball hard, the effect of the incline is reduced, however, it moves away from the hole when the ball does not hole-in. Conversely, a weak hit increases the effect of the slopes. Therefore, by investigating the golfers’ aim, movement, and recognition of inclines, issues pertaining to steep putting may be understood.

Karlsen and Nilsson [2] described the factors that influence putting results in the chronological order of green reading (60%), aiming and stroke (34%), and green inconsistencies (6%). Green reading involves actively moving around and perceiving the relative borrow or environmental characteristics that will cause the ball to deviate from a straight trajectory. Several studies have investigated gaze behavior during green reading [3]. According to the van Lier et al. [4], as the slope became steeper, experts had more fixations on the high side of the hole before the putt. Although many studies in the field of sports psychology have focused on the putting task [5–12], few studies have focused on slopes [4, 13, 14].

Hasegawa et al. [14] investigated golfer’s perception and movement at 0- and 0.4-degree slopes and found that many amateurs could not properly perceive the presence or absence of a slight slope. In other words, most amateurs made more visual-somatosensory errors than professionals did and found that their slope perception influenced subsequent performance. In contrast, most professionals could accurately detect subtle differences in slopes. The results of Hasegawa et al. also suggest that professional golfers refine their cognitive motor processes during challenging situations, whereas amateurs have underdeveloped cognitive motor processes, as shown in previous studies [15]. In other words, superior motor performance in professionals is characterized by inhibition of task-irrelevant cognitive-motor processes and facilitation of essential perceptual-motor control processes during motor preparation [16, 17]. Furthermore, the superior performance of skilled golfers is characterized by dynamic neuromotor processes related to attentional focus [18, 19]. These studies may suggest that “amateur side” involves poor environmental recognition and motor control issues, such as immature visual-somatosensory perception that doesn’t properly sense inclines, or poor recognition owing to insufficient attention on the environment.

To address these issues, we experimentally confirm the phenomenon known as the amateur side in golf. The slope of the putting surface required for our experiment is 1 to 3 degrees, which significantly influences the ball’s roll, instead of slight inclines that are difficult to detect. Empirically, a slope of 1 degree is commonly experienced on golf courses, and 3 degrees is classified as the steepest slope on actual greens. The research question of the previous study [14] was whether golfers can detect subtle differences of slopes, but it did not clarify the issue on the amateur side. To experimentally examine the ball miss to the front-lower side of the hole, preparing the conditions in which most people can clearly recognize the presence of slopes is necessary, such as a relatively large break in the ball trajectory. Therefore, the recognition of the slopes’ steepness remains unclear. We hypothesized that amateurs have lower aims and launch angles than professionals, the same as the results of Hasegawa et al. [14]. We also hypothesized that the correlation between the angle of the launched ball and the impact speed of the putter head in amateurs is lower than those of professionals, because amateurs’ movements have a larger variability than those of professionals, as has been revealed in many previous studies [9, 14, 20]. In addition, we set a task, which used a miniature platform to investigate how golfers recognized the steepness of the slopes. We assumed that amateurs, compared to professionals, underestimate the steepness and that the recognition of the slopes and the aim (angle) are correlated.

The first objective of this study was to examine whether a front-down bias toward the hole could be experimentally observed in golfers’ putting. The second objective was to determine, by measuring aims and movements, why golfers, especially amateurs, putt poorly on steep slopes. The third objective was to investigate how golfers recognized the steepness and the relationship between their aim and their slope recognition. This may provide an interesting perspective for researchers, as the issues suggested by the amateur side occurring on real golf courses have not been studied before.

## Materials and methods

### Participants

This study included 12 tour professionals (participating in JGTO or JLPGA tournaments; 7 males, 5 females) and 12 amateur golfers (6 males, 6 females) whose average ages were 35.8 ± 6.1 and 47.5 ± 7.4 years, respectively, with golfing experiences of 24.2 ± 6.9 and 19.3 ± 9.1 years. The amateurs were intermediate players with an average score of 88.3 ± 2.5 for 18 holes (handicap around 15). All participants were right-handed golf players with normal or corrected-to-normal vision. All participants provided written informed consent after a thorough explanation of the study. All experimental procedures were approved by the Ethics Committee of Iwate University and conformed to the principles of the Declaration of Helsinki. The recruitment period for this study was from February 1, 2019 to March 20, 2019.

### Task and apparatus

**Fig 1:**
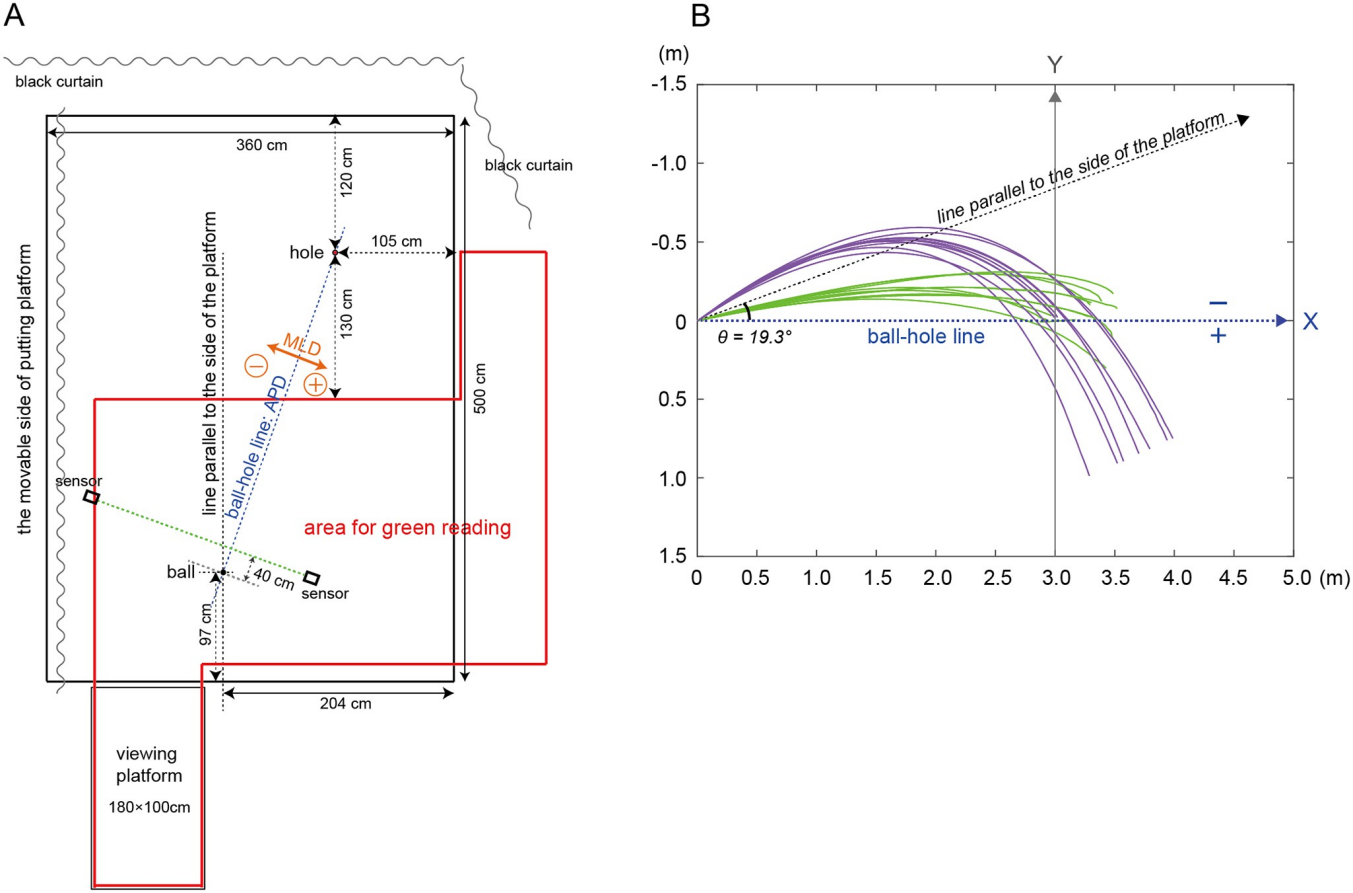
Experiment setting and an example of all ball trajectories for a professional golfer in both conditions. (A) represents the pattern diagram of the experiment setting, while (B) shows the definition of measurements and an example of all ball trajectories for a professional golfer in both conditions. The experiment area where the putting platform was set was surrounded by curtains. The area surrounded by the red line indicates where participants could move freely to read the green. Two sensors were placed so that the shutter goggles would be activated when the ball passed the line 40 cm in front of where it was set. APD indicates the anteroposterior direction, while MLD indicates the mediolateral direction. The ball-hole line was approximately 19.3 degrees to the line parallel to the side of the platform and was to the right of the ball in the hitting direction. The clubface orientation of all phases was calculated as 0 degrees when the putter face was perpendicular to the ball-hole line. The green lines indicate the ball trajectory for the 1-degree condition, and the purple lines indicate the ball trajectory for the 3-degree condition. In addition, in the 3-degree conditions, it was not possible to identify the stopping position of the ball due to the size limitation of the putting platform. Therefore, the end of the plot of the ball’s trajectories represent the edge of the platform. Coordinates (0,0) indicate the initial ball position, and (3,0) indicate the center of the hole.

The task included a 3 m putting distance under 1- and 3-degree conditions. We set these two conditions because the task difficulty depends on the degree of inclination. van Lier et al., [4] also set a slope of 3°, which is the steepest slope among the greens of general golf courses. In addition, 1° was selected, assuming a typical level of slope in general golf courses. For both conditions, a slice line (tilted to the right relative to the striking direction) was set, which follows the empirical finding that most golfers are not proficient in slice lines compared to hook lines (right-to-left line). Participants hit 10 balls in each condition without visual feedback of outcomes, and their goal was to get a ball into a hole 10.8 cm wide (the same size as that in an actual golf green) in one putt. Additionally, the participants were asked to hit the ball with the intention that there would be a next play, just like playing on a golf course. In other words, participants were asked to hit the ball at a distance that would allow them to make a hole-in on their second shot. Furthermore, the participants were asked to indicate their aim point from which the ball would be launched (i.e., aim direction) before putting. Participants did not receive any explicit slope or distance information. Immediately after playing each condition, participants were asked to reproduce the slope they performed on using the miniature platform (Fig 1).

The artificial turf used in this experiment was designed for practice (5.0 m long × 3.6 m wide; Superbent, Newtons Inc., Kochi, Japan). The stimp rating, which indicates the speed of a putting green, was approximately 12 ft and classified under the fast category. The putting platform was a metal frame on a grid with robust wooden boards attached with an artificial turf. In addition, this platform was designed to be operable on one side (5 m long) using electric winches. The ball-to-hole line was approximately 19.3° to the line parallel to the side of the platform and to the right of the ball in the hitting direction (see Fig 1). In addition, owing to the limited size of the putting platform (5.0 m × 3.6 m), capturing the final ball position under the 3-degree condition was not possible. In the green reading area for slope perception, the participants were allowed to approach the hole up to 1.3 m away. The participants were also allowed to go up to 2.8 m behind the ball.

All participants wore instant shielding goggles (AO-FOS; Applied Office Co., Ltd., Tokyo, Japan), which limited their field of view to 40 cm in front of the ball. The system was shielded when a ball crossed the light between the photoelectric sensors. This was necessary to prevent learning adjustments during the trials to confirm perceptions and strategies. The goggle lens was transparent when there was no shutter, and the field of view was clear from up to 6 ft away. The participants’ hearing was unobstructed so they could hear the impact of the ball. However, the participants could not hear the ball rolling because of the artificial turf design. Further, participants did not know whether their putts had made it into the hole or not, as the hole was designed to make no noise when the ball entered it.

**Fig 2:**
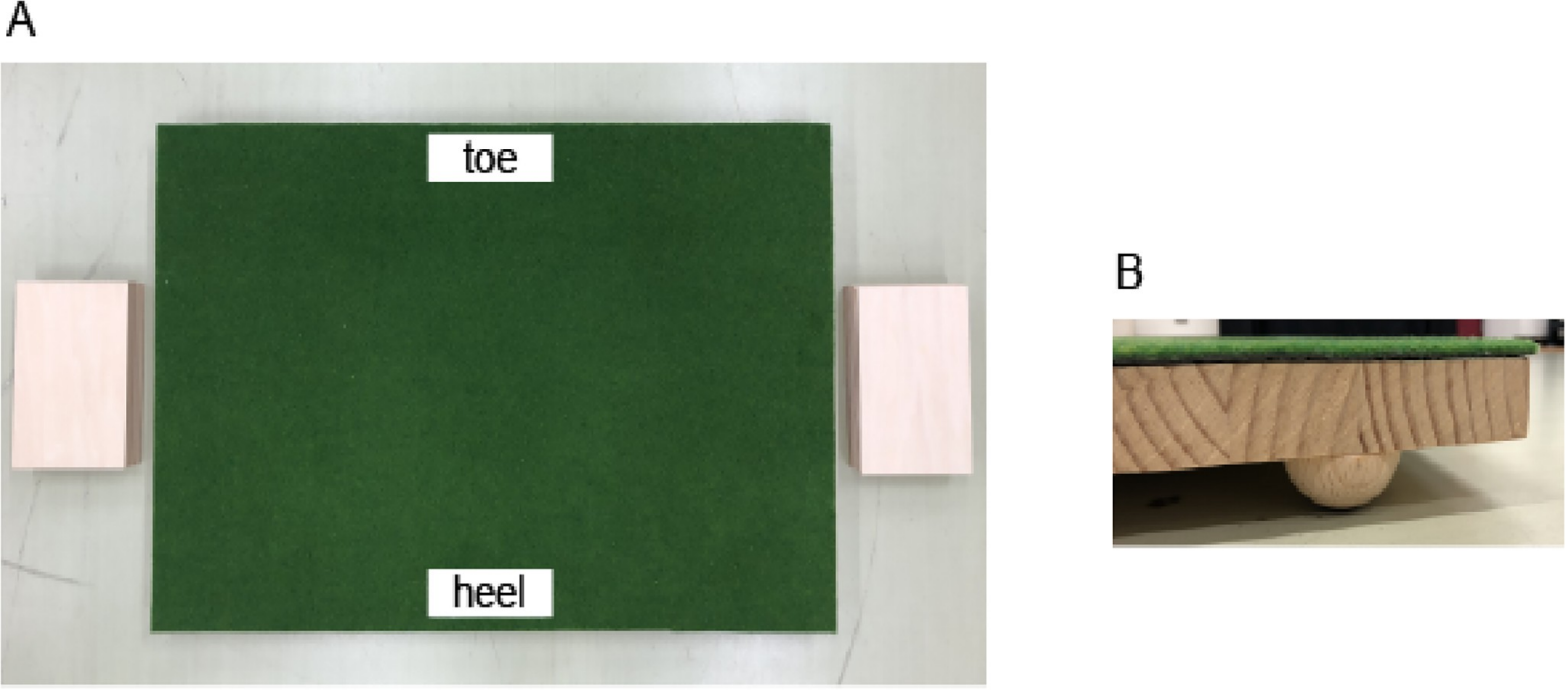
Miniature platform used in the slope reproduction task. (A) shows the actual miniature platform (600 mm long x 910 mm wide x 300 mm high + 25 mm with feet). There are 12 boards (150 mm long x 225 mm wide x 4 mm high) on each side. (B) shows a part of it from the side view. The semi-circular feet (50 mm in diameter) were attached to the four corners of the platform. It was designed so that the slope could be adjusted by 0.5 degrees when one plate was inserted on both the left and right sides.

Based on previous research [21, 22], we asked participants to reproduce the slopes using a miniature putting platform after their trials,. For the miniature platform used in the slope reproduction task, there were 12 boards on each side of the platform. By placing these boards on the toe or heel side, the participants were able to create uphill and downhill slopes (see Fig 2). The size of the miniature platform to measure the participants’ perception was 600 mm long x 910 mm wide x 300 mm high + 25 mm with feet. The semi-circular feet (50 mm in diameter) were attached to the four corners of the platform with 12 boards (150 mm long x 225 mm wide x 4 mm high) on each side. It was designed so that the slope could be adjusted by 0.5 degrees when one plate was inserted on both the left and right sides.

The aim direction, putter-head kinematics, and ball trajectories were recorded using nine optical motion capture cameras (Prime13, OptiTrack Japan, Ltd., Tokyo, Japan) operating at 240 Hz. Twelve-millimeter reflective markers were attached to the toe, heel, and neck of the putter head. Additionally, a reflective sheet was attached to the ball to capture its trajectory [14]. The root mean square error of the static and dynamic calibrations was < 1 mm for all sessions. All participants used the same putter (SB-01HB; PRGR Corp., Yokohama, Japan) and balls (Srixon Z-Star XV; Dunlop Sports Co. Ltd., Hyogo, Japan).

### Procedure

Considering the order effect, the 1- and 3-degree conditions were in random order and counterbalanced. In each condition, the participants practiced with 10 balls in the waiting room to familiarize themselves with the artificial turf. However, during the familiarization sessions, the surface was flat, without slope. In the familiarization session, participants were asked to wear shielding goggles. However, the participants’ field of view was unobstructed after hitting. The participants then moved to the experimental area with the researcher and received the following instructions while viewing the actual putting platform: “Please try to putt 10 balls here. Your goal is to get the ball into the hole with one putt. You do not actually hit the second putt in this trail, but just like playing on the actual course, please imagine that you would need to make a hole in the next putt if you do not make a hole in one. That is, please try to get the ball as close to the hole as possible. Feel free to move around for the next three minutes and read the putting line. Please remember the slope on which you stand during this trial.”

Subsequently, the green reading area was explained to the participants. Three minutes later, the participants put on the instant shielding goggles. They were then instructed on how to set the ball and place it in position. Afterwards, they crouched behind the ball towards the target, as they would in actual play, and indicated their intended ball launch direction. The researcher moved the aim point marker while following the participants’ instructions and adjusted it until they thought it was in the correct position. The researcher recorded the marker position and then attached a circular white sticker (0.8 cm diameter) to the artificial turf at the same position as the aim point marker [14]. In an actual golf green, golfers find an aim point in the direction they want to launch the ball and set it up towards this spot. Because the artificial turf did not have any discoloration (uniformly green), the participants could be prevented from losing sight of the aim direction during the set-up when the white sticker was attached. Subsequently, the participants practiced only once to familiarize themselves with their inhibited field of view immediately after hitting the ball. The participants were also informed that there was no time limit for hitting.

The participants repeated this process 10 times after the practice phase when they indicated their aim to hit the ball. After the condition trial, they moved to the waiting area and tried the slope reproduction task. The participants were asked to place the number of boards on either the toe or heel side to reproduce the slope in the previous condition. There were no time limitations. Participants took a 15-minute break, after the slope reproduction task, and then attempted another condition.

### Dependent variables

#### 1. Kinematics

All digitized data were smoothed with a fourth-order Butterworth filter (5-Hz cut-off) based on the root mean square error of the residuals between the original and smoothed data [23, 24]. The aim direction that the ball would be launched was calculated as the angle with a line connecting the ball and the center of the hole of 0° (see Fig 1). The putterface orientation of the setup immediately before hitting (address angle) was calculated as 0° when the putter face was perpendicular to the ball-hole line. The peak speed of the putter head was calculated by combining the velocities along the x- and y-axes; ball travel distance is highly dependent on peak velocity [25–27]. In addition, the ball launch angle was determined and calculated using the direction of the ball after hitting, that is, the average angle for 0.1 s after ball impact. For all angles, negative values indicated the left side of the ball-hole line and positive values indicated the right side of the ball-hole line (see also Fig 1). In addition, the speed (i.e., combining the velocities along the x- and y-axes) and position of the ball were calculated when crossing the coordinate line at the left end of the hole.

#### 2. Slope reproduction task

The number of boards inserted in the miniature platform was counted and recorded, along with their position (toe or heel) (see also Fig 2).

### Statistics

The relationships between the two groups (professional and amateur) and the two types of conditions (1-degree and 3-degree) in terms of the average aim angle, address angle, launch angle, peak speed, ball speed near the hole, and ball position when crossing the coordinate line at the left end of the hole were examined using a two-factor mixed design analysis of variance (ANOVA). Even though MANOVA can be effectively used in this case to consider the relationship between independent variables while trying to find differences between professionals and amateurs, we examined each independent variable separately based on our hypothesis, as in previous studies. In particular, the angle in each phase are important variables [28]. Therefore, using ANOVA gives us the advantage of comparing the results with those of similar studies [13, 14]. Additionally, Pearson’s correlation analysis was performed to identify the relationship between the ball launch angle and peak speed for each group. Welch’s t-test was performed for each condition to verify the differences between the groups regarding the ball-launch angle and ball velocity of the hole-in trials.

To assess the participants’ slope reproduction task, we classified their estimates into three categories according to the number of boards inserted by the participants: under (i.e., less than the actual angle), equal (i.e., the same as the actual angle), and over (i.e., more than the actual angle) estimation. It is more appropriate to qualitatively evaluate whether participants recognized the inclines properly than to quantitatively evaluate the average number of boards used by the participants. The frequency in each category was counted, and chi-square tests were conducted for each condition. In addition, to examine the relationship between the degree of achievement in the reproduction task and the aiming angle, we conducted Spearman’s rank correlation test on the number of boards inserted by participants and ranking of the aiming angle (1 = smallest, 24 = largest).

We calculated the “f” and “d” values as effect-sizes indices for the ANOVAs [29] and Cramer’s V values as effect-size indices for the chi-squared tests. According to Cohen’s conventions [30], effect sizes were reported as small (f = 0.10, d = 0.20, Cramer’s V = 0.10), medium (f = 0.25, d = 0.50, Cramer’s V = 0.30), and large (f = 0.40, d = 0.80, Cramer’s V = 0.50). All data were analyzed using PASW Statistics (ver. 18.0; IBM Japan Ltd., Tokyo, Japan).

## Results

### Kinematics

#### 1. Aim direction

**Fig 3:**
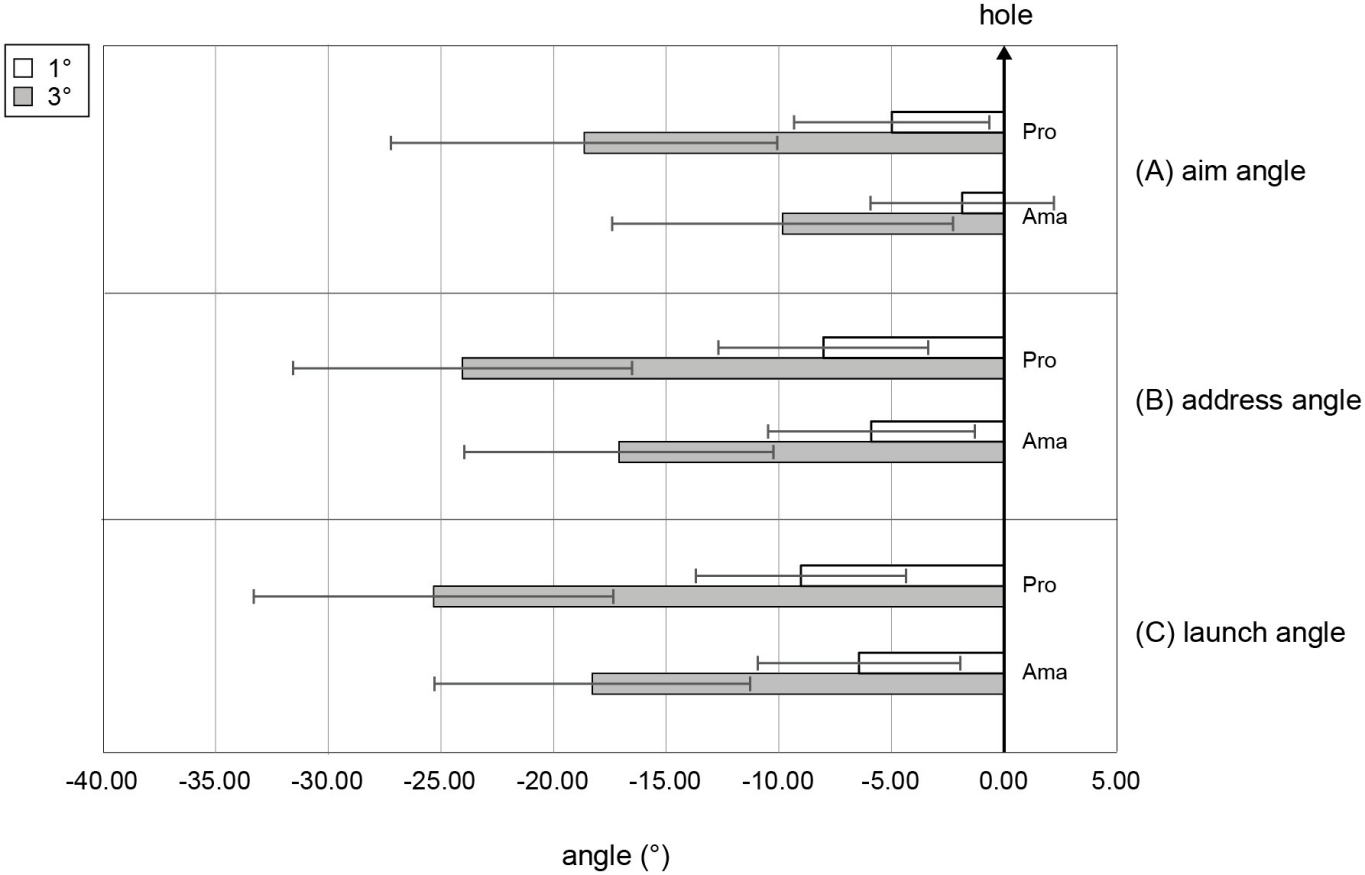
Average angles of each phase (A–C) in the 1- and 3-degree conditions. Angle 0 refers to the center of the target. Negative values indicate the position to the left of the target’s center, while positive values indicate the position to the right of the target’s center. All error bars indicate ± 1SD.

The results of the two-factor ANOVA for aim angle (Fig 3A) showed that the interaction effect (group × condition) was not significant. However, the main effects of group (*F*_1,22_ = 14.25, *p* = .001, *f* = 0.80, 1 − *β* = 0.99) and condition (*F*_1,22_ = 53.00, *p* = 2.72 × 10^−7^, *f* = 1.55, 1 − *β* = 1.00) were significant. In other words, the aiming angle of the professional group was more to the left than that of the amateur group, regardless of the condition, and the aim angle in the 3-degree condition was more to the left than that in the 1-degree condition, regardless of skill level.

#### 2. Address direction

The results of the two-factor ANOVA for address angle (Fig 3B) showed that the interaction effect was not significant. However, the main effects of group (*F*_1,22_ = 4.74, *p* = .040, *f* = 0.46, 1 − *β* = 0.70) and condition (*F*_1,22_ = 105.61, *p* = 7.33 × 10^−10^, *f* = 2.19, 1 − *β* = 1.00) were significant. That is, the address angle of the professional group was more to the left than that of the amateur group, regardless of the conditions, and the aim angle in the 3-degree condition was more to the left than that in the 1-degree condition, regardless of skill level.

#### 3. Launch direction

The results of the two-factor ANOVA for launch angle (Fig 3C) showed that the interaction effect was not significant. However, the main effects of group (*F*_1,22_ = 5.05, *p* = .035, *f* = 0.48, 1 − *β* = 0.74) and condition (*F*_1,22_ = 106.38, *p* = 6.86 × 10^−10^, *f* = 2.20, 1 − *β* = 1.00) were significant. In other words, the launch angle of the professional group was more to the left than that of the amateur group, regardless of the conditions, and the aim angle in the 3-degree condition was more to the left than that in the 1-degree condition, regardless of the skill level.

#### 4. Peak speed of putter head

**Fig 4:**
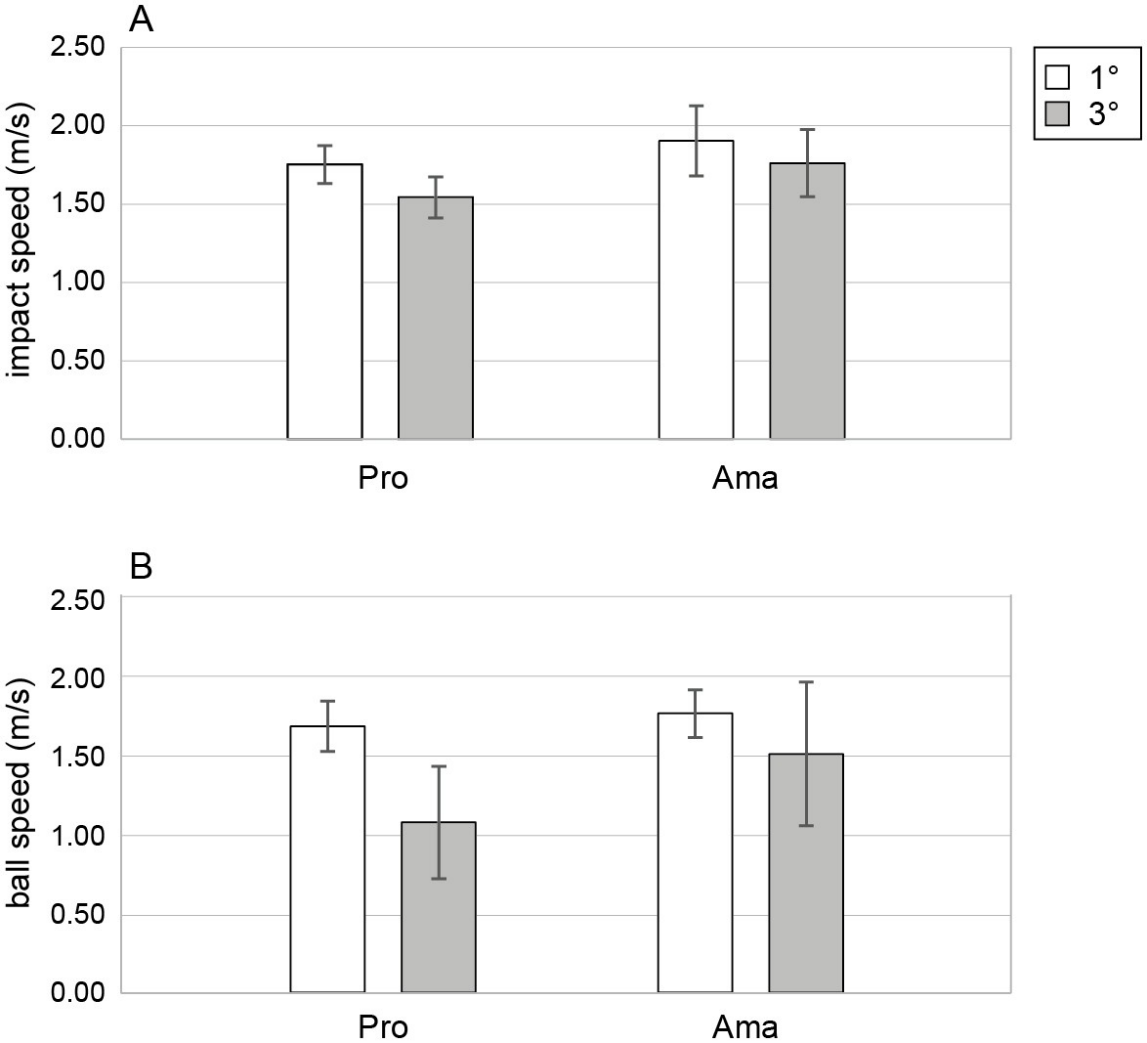
Averages of peak speed of putter-head and ball speed near the hole in the 1- and 3-degree conditions. (A) indicates the averages of peak speed of putter head. (B) indicates the averages of ball speed near the hole. All error bars indicate ± 1SD.

The results of the two-factor ANOVA for the peak speed (Fig 4A) showed that the interaction effect was not significant. However, the main effects of group (*F*_1,22_ = 6.87, *p* = .016, *f* = 0.56, 1 − *β* = 0.86) and condition (*F*_1,22_ = 71.83, *p* = 2.24 × 10^−8^, *f* = 1.81, 1 − *β* = 1.00) were significant. In other words, the peak speed of the professional group was lower than that of the amateur group, regardless of the conditions, and the aim angle in the 3-degree condition was lower than that in the 1-degree condition, regardless of the skill level.

#### 5. Ball speed

The two-factor ANOVA results for ball speed (Fig 4B) revealed a significant interaction effect (*F*_1,22_ = 5.33, *p* = .031, *f* = 0.49, 1 − *β* = 0.63). Simple effects testing indicated that the ball speed of the professional group in the 3-degree condition was lower than that of the amateur group (*F*_1,22_ = 6.77, *p* = .002, *f* = 0.55, 1 − *β* = 0.84). In addition, the ball speed in the 3-degree condition was lower than that in the 1-degree condition for both the professional (*F*_1,22_ = 31.78, *p* = 1.14 × 10^−5^, *f* = 1.20, 1 − *β* = 1.00) and amateur groups (*F*_1,22_ = 5.63, *p* = .027, *f* = 0.51, 1 − *β* = 0.91).

#### 6. The relationship between the peak speed and the launch angle

**Fig 5:**
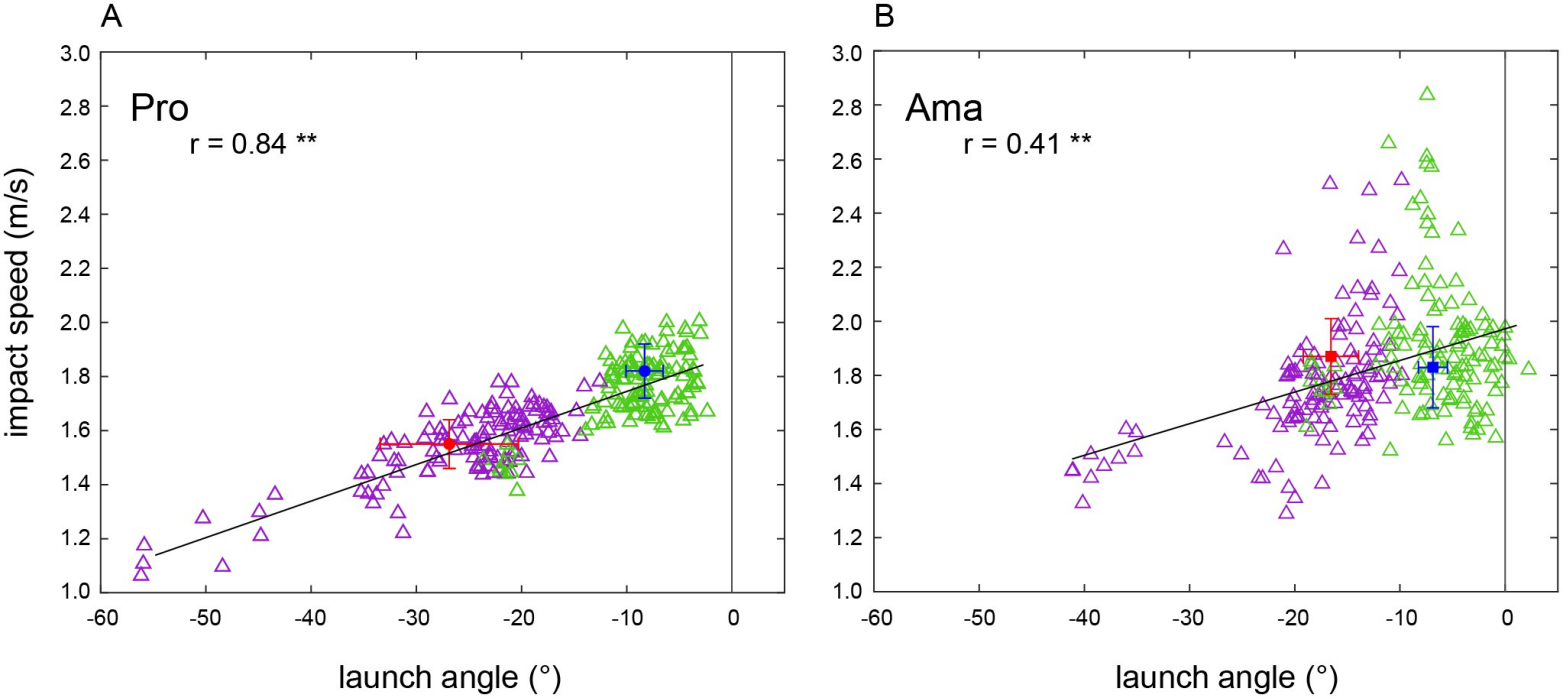
Relationship between ball launch angle and peak speed of all participants’ trials and hole-in trials. The plots of all 240 trials involved ball launch angle and peak speed for the 12 professionals (A) and 12 amateurs (B). The blue and red plots show the averages and SD (± 1SD) of the hole-in trials in the 1- and 3-degree conditions, respectively.

The results of Pearson’s correlation analysis indicated a positive correlation between the peak speed of putter head and the launch angle of the ball (*r* = 0.84, *p* < .01; Fig 5A). Additionally, the two variables were positively correlated for the amateur datasets (*r* = 0.41, *p* < .01; Fig 5B).

#### 7. Ball position near the hole

Table 1 lists the ball position when crossing the coordinate line at the left end of the hole. The results of the two-factor ANOVA for the peak speed near the hole, interaction and main effects were not significant.

**Table 1:** The ball position when crossing the coordinate line at the left end of the hole.

	1 degree		3 degree	
group	Pro	Ama	Pro	Ama
average	-0.33	-0.42	-0.15	-0.27
sd	0.40	0.72	0.27	0.40

The unit is (m).

#### 8. Hole-in trials

Fig 5 shows the average ball launch angles and peak speeds of the hole-in trials for each condition. Regarding the the average ball launch angle in the hole-in trials, Welch’s t-test revealed significant differences between professionals and amateurs in both conditions (1-degree: *t*_28_ = 3.32, *p* = .003, *d* = 0.53, 3-degree: *t*_23_ = 1.07 × 10^−5^, *d* = 0.76). Regarding the peak speed of putter head in the hole-in trials, Welch’s t-test revealed a significant difference between professionals and amateurs in the 3-degree condition (*t*_10_ = 5.73, *p* = 1.91 × 10^−4^, *d* = 0.88). However, there was no significant difference in 1-degree condition. In other words, the professional’s launch angles were to the left of those of the amateurs in both conditions, and the professional’s ball speed was lower than that of the amateurs only in the 3-degree condition.

#### 9. Slope reproduction task

**Fig 6:**
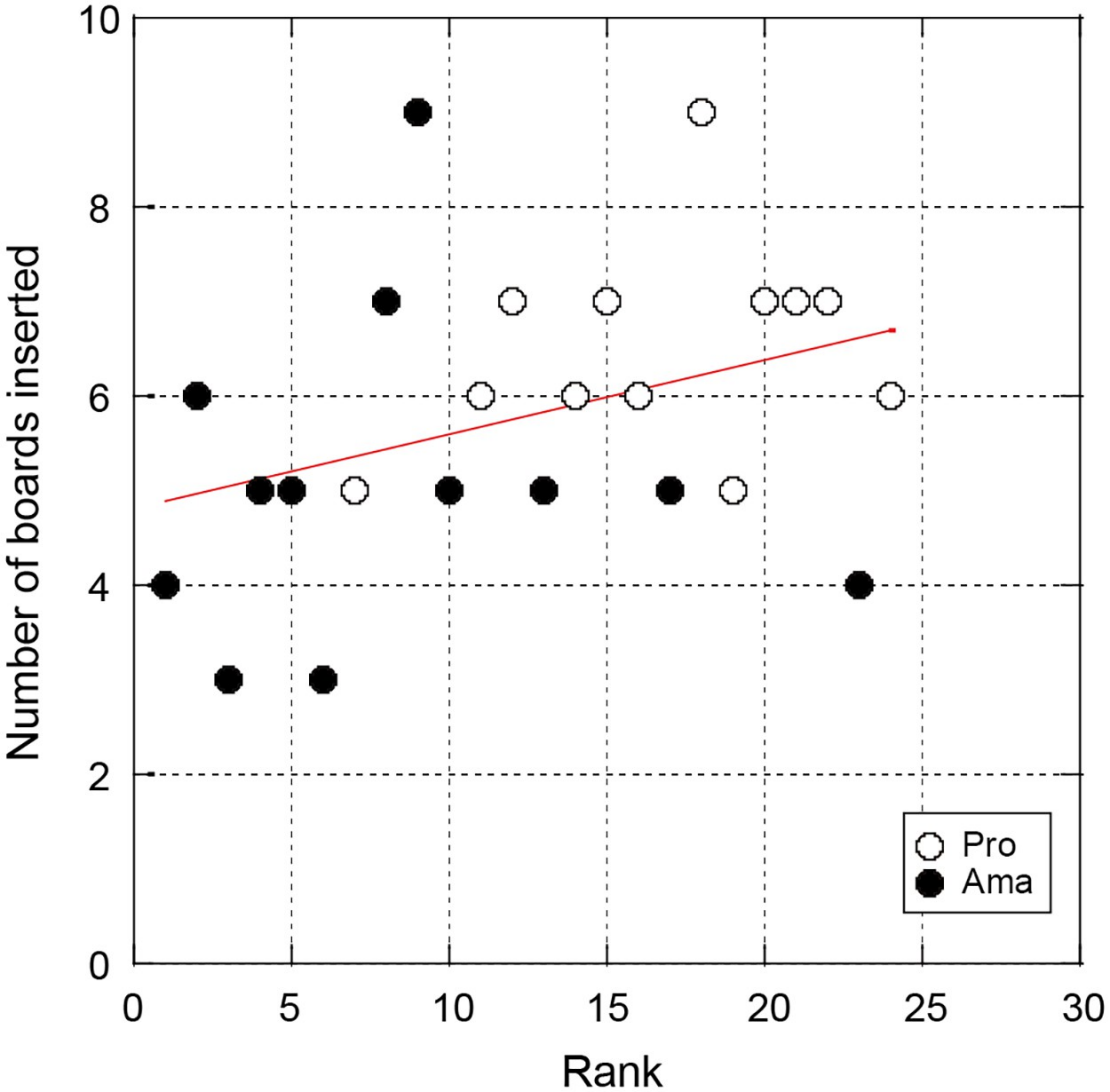
Relationship between the number of boards inserted by participants and the aiming angle ranking in the 3-degree condition. Aiming angles were ranked based on their values (1 = smallest, 24 = largest). The vertical axis shows the number of plates inserted by the participant after the trial.

Tables 2 and 3 show the frequencies of the three categories under the 1- and 3-degree conditions in both groups, respectively. No participant placed a board on the toes during the task. Chi-squared tests revealed significant differences in the 1-degree condition between the two groups (*χ*[2] = 9.16, *p* < .05, *Cramer*^′^*sV* = 0.62). Residual analysis indicated that the number of professionals was significantly higher than the number of amateurs in the equal category, and the number of amateurs was significantly higher than the number of professionals in the under category. Additionally, chi-square tests revealed a significant difference in the 3-degree condition between the two groups (*χ*[2] = 8.26, *p* < .05, *Cramer*^′^*sV* = 0.59). Residual analysis confirmed that the number of amateurs was significantly higher than the number of professionals in the under category. The Spearman’s rank correlation test on the number of boards and the ranking of aiming angle revealed a significant trend on the 3-degree condition (*r* = 0.36, *p* = .083; Fig 6).

**Table 2:** The frequency of three categories under 1-degree conditions.

	under		equal		over	
Pro	1	▽	9	△	2	
Ama	7	△	2	▽	3	

▽△ indicate the result of residual analysis.

**Table 3:** The frequency of three categories under 3-degree conditions.

	under		equal		over	
Pro	2	▽	4		6	
Ama	9	△	1		2	

▽△ indicate the result of residual analysis.

## Discussion

This is the first study to experimentally examine the problem occurring on an actual golf course, known as the “amateur side”. We found significant differences between the groups (Fig 3) in the aim, address, and ball-launch direction. In other words, the amateurs’ directions were smaller than those of the professionals in all phases, which suggests that the amateurs aimed more to the right than the professionals did. In the slope reproduction task conducted immediately after each condition to measure how the participants perceived the slopes, most amateurs underestimated the severity of the slope in both conditions (Tables 2 and 3). Additionally, a weak correlation was found between golfers’ aim and slope recognition.

Although the magnitude of the slope setting differs, our results are similar to those of Hasegawa et al. [14], which also suggests that amateurs have a poor slope perception. Additionally, Hasegawa et al. [14] reported that the perception of the slope is an important variable that explains the final ball position. Therefore, we propose that the aim directions of the amateurs were smaller than those of the professionals owing to their underestimation of the slope and that this is the main reason amateurs for their poor performance while putting on sloped greens. Because the address and ball-launch direction are based on the aim direction [14], there is inevitably a difference between the two groups in these two angles. In addition, in the 3-degree condition, a weak correlation was found between the number of boards inserted by participants in the reproduction task and the aiming direction. In the present study, it was not possible to directly measure participants’ sensation during the task, and measurements were taken after 10 trials. Therefore, some participants might have responded to the tasks with their somatosensory memory of the slopes gained during the trial. However, we believe that our approach was valid, as this type of post-mortem investigation has been used in previous studies [12, 21, 22].

Despite putting at the same target distance, the peak speed of amateurs was higher than that of professionals (Fig 4). We speculate that amateurs chose smaller launch directions because they underestimated the slopes. As a result, their aim directions were smaller than those of the professionals. In this case, it is understandable that they would need a higher peak speed than the professionals. However, there could be no such reasonable relationship between launch direction and impact speed. An analysis of the correlation between the peak speed of the putter head and ball launch angle revealed the second reason for amateurs having poor performance while putting on sloped greens. We found a strong positive correlation between the ball launch angle and peak speed of putter head for professionals, whereas amateurs had a weak positive correlation (Fig 5). This suggests that amateurs are unable to perform the peak speed of the putter head according to the ball-launch angle, or vice versa.

Moreover, the analysis of ball speed near the hole indicated that the ball speed of amateurs was faster than that of professionals (Fig 4B). This implies that if the ball missed the hole, it moved farther from the hole. Additionally, regarding the hole-in trial, amateurs had a faster ball speed near the hole in the 3-degree condition, and the ball launch angle was smaller than that of the professionals under both conditions (Fig 5). In other words, amateurs were more likely to miss the putt in hole-in trials than were professional. In this study, participants were not required to hit the next putt when the ball did not enter the hole; however, like an actual golf play, the task had a constraint. That is, the participant had to imagine that they must make a hole in with their next putt if they do not make a hole-in one. This constraint should prevent the golfers from hitting balls too hard, as in a real game. To obtain a good score, appropriate action strategies are necessary; however, amateurs’ lacked action strategies compared to professionals.

Contrary to our expectations, most professional trials ended on the lower front side towards the target just like those of amateurs (Table 1). In other words, their putts often missed on the amateur side. However, regarding the slope reproduction task, most professionals were able to properly perceive the slopes, especially in the 1-degree condition. Nevertheless, the fact that most of the professional trials were bent in front of the hole may be due to the experimental setting. Our setting was different from an actual golf course green (i.e., artificial turf); therefore, the ball trajectory may have bent more than they imagined from their experience. Familiarization sessions were held in the waiting room; thus, the participants had never seen the ball trajectories on the platform used in the measurement. We assume that this may have had a significant effect on the results.

This is the first study to experimentally examine the problem occurring on an actual golf course, known as the “amateur side”, and although some issues remain, we were able to show some noteworthy results. As for the limitations of this study, first, to examine the participants’ recognition of the sloped green, we asked participants to use boards that varied by 0.5 degrees each. For this reason, it may not be possible to clarify a significant relationship between the sense of inclination and aiming direction significantly. To examine these relationships in the future, researchers should devise ways to further distinguish the participants’ responses. Redundancy was observed in launch angle and peak speed of putter head even in the hole-in trials. Further, as mentioned above, we found that the launch angle and the peak speed of professionals were better than those of amateurs; however, we did not know what the optimal solution was. Third, we measured the slice line where the ball bent from left to right, but we also had to verify the hook line where the ball break from right to left. Fourth, our sample size was small, future studies should increase the sample size and examine these issues. Fifth, owing to limitations of the experimental system, confirming the final ball position was not possible, especially in the 3-degree condition. However, this issue could be compensated by measuring ball speed and position around the hole as an alternative indicator. Although we did not set a standard flat condition, our results can be compared with the results of Hasegawa et al. [14] including the 0-degree condition and other studies that used flat situations.

## Conclusion

We attempted to clarify the bias of the front-lower side towards a hole in golf putting, which is often seen on the sloped greens of an actual golf course. We compared the putter head and ball kinematics of professionals and amateurs in the 1- and 3-degree conditions with the left-to-right (slice) line. In addition, we examined how golfers recognized slope conditions using a slope-reproduction task. In this study, we experimentally confirmed the bias to the front-lower side of the hole, which is also called the amateur side. In addition, we identified two reasons for putting inefficiency, especially in amateur golfers. First, the amateurs’ putter head angles were more to the right than the professionals’ putter head angles in each phase, even though tilted to the right relative to the striking direction. The slope reproduction task also highlighted that many amateurs underestimated the severity of slopes on both slopes, which is the primary reason for their poor putting on steep slopes. Second, regarding the relationship between the peak speed of the putter head and ball launch angle, a strong positive correlation was found for professionals, whereas a weak correlation was found for amateurs. This implies that amateurs are unable to optimize the peak speed according to the ball launch angle, or vice versa. We hope that our results assist in improving golfing athletes’ and coaches’ skills. As practical indications of this research, we suggest the development of applications to improve visual sensory processes, such as neurofeedback [31], biofeedback training [32], and imagery training, such as strengthening the imagery of the ball’s trajectory.

## References

[1] Karlsen J., Smith G., and Nilsson J. The stroke has only a minor influence on direction consistency in golf putting among elite players. J Sports Sci. 2008 Feb;26:243–50. doi: 10.1080/02640410701530902 17917952

[2] Karlsen J, Nilsson J. Distance variability in golf putting among high skilled players: The role of green reading. Int J Sports Sci Coach. 2008 Jul;3:71–80. doi: 10.1260/174795408785024333

[3] Ziv G, Lindor R. Gaze Behavior in golf putting—A review. Int. J. Golf Sci. 2019 Sep;7(2):https://www.golfsciencejournal.org/article/10140-gaze-behavior-in-golf-putting-a-review.

[4] van Lier W, van der Kamp J, Savelsbergh GJP. Gaze in golf putting: Effects of slope. Int J Sport Psychol. 2010 Apr;41(2):160–176.

[5] Bell JJ, Hardy J. Effects of attentional focus on skilled performance in golf. J Appl Sport Psychol. 2009;21(2):163–177. doi: 10.1080/10413200902795323

[6] Cooke A, Kavussanu M, McIntyre D, Boardley ID, Ring C. (2011). Effects of competitive pressure on expert performance: Underlying psychological, physiological, and kinematic mechanisms. Psychophysiology. 2011 Aug;48(8):1146–1156. doi: 10.1111/j.1469-8986.2011.01175.x 21265862

[7] Craig CM, Delay D, Grealy MA, Lee DN. Guiding the swing in golf putting. Nature. 2000 May;405:295–296. doi: 10.1038/35012690 10830947

[8] Delay D, Nougier V, Orliaguet J, Coello Y. Movement control in golf. Hum Mov Sci. 1997 Oct;16(5):597–619. doi: 10.1016/S0167-9457(97)00008-0

[9] Hasegawa Y, Okada A, Fujii K. A sense of distance and movement characteristics of golfers tested without visual feedback of outcomes: Is a putt that feels subjectively good also physically good? Front Sports Act Living. 2022 Oct;4:987493. doi: 10.3389/fspor.2022.987493 36385781 PMC9640950

[10] Tanaka Y, Sekiya H. The influence of audience and monetary reward on the putting kinematics of expert and novice golfers. Res Q Exerc Sport. 2010 Dec;81(4):416–424. doi: 10.1080/02701367.2010.10599702 21268465

[11] Wilson M, Smith NC, Holmes PS. The role of effort in influencing the effect of anxiety on performance: Testing the conflicting predictions of processing efficiency theory and the conscious processing hypothesis. Br J Psychol. 2007 Aug;98:411–428. doi: 10.1348/000712606X133047 17705939

[12] Witt JK, Linkenauger SA, Bakdash JZ, Proffitt DR. Putting to a bigger hole: Golf performance relates to perceived size. Psychon Bull Rev. 2008 Jun;1(3):581–585. doi: 10.3758/pbr.15.3.581 18567258 PMC3193943

[13] Dias G, Couceiro MS, Barreiros J, Clemente FM, Mendes R, Martins FM. Distance and slope constraints: Adaptation and variability in golf putting. Motor control. 2014 Jul;18(3):221–243. doi: 10.1123/mc.2013-0055 24280087

[14] Hasegawa Y, Okada A, Fujii K. Skill differences in a discrete motor task emerging from the environmental perception phase. Front Psychol. 2021 Oct;12:697914. doi: 10.3389/fpsyg.2021.697914 34659013 PMC8517186

[15] Chen TT., Wang KP., Huang CJ., Hung TM. Nonlinear refinement of functional brain connectivity in golf players of different skill levels. Scientific Reports. 2022 Feb;12:2365. doi: 10.1038/s41598-022-06161-3 35149719 PMC8837743

[16] Wang KP., Chengc MY., Chend TT., Huange CJ., Schacka T., Hungf TM. Elite golfers are characterized by psychomotor refinement in cognitive motor processes. Psychol Sport Exerc. 2020 Sep;50:101739. doi: 10.1016/j.psychsport.2020.101739

[17] Wang KP., Cheng MY., Chen TT., Lin KH., Huang CJ., Schack T., et al. Successful motor performance of a difficult task: Reduced cognitive-motor coupling. Sport Exerc Perform Psychol. 2022 Nov;2:174–184.

[18] Wang KP., Cheng MY., Chen TT., Chang YK., Huang CJ., Feng J., et al. Experts’ successful psychomotor performance was characterized by effective switch of motor and attentional control. Psychol Sport Exerc. 2019 July;43:374–379. doi: 10.1016/j.psychsport.2019.04.006

[19] Wang KP., Frank C., Tsai YY., Lin KH., Chen TT., Cheng MY., et al. Superior performance in skilled golfers characterized by dynamic neuromotor processes related to attentional focus. Front Psychol. 2021 Feb;16:633228. doi: 10.3389/fpsyg.2021.633228 33664700 PMC7921727

[20] Tanaka H, Iwami M. Estimating putting outcomes in golf: Experts have a better sense of distance. Percept Mot Skills. 2018 Apr;125:313–328. doi: 10.1177/0031512518754467 29357738

[21] Lee Y, Lee S, Carello C, Turvey MT. An archer’s perceived form scales the “hitableness” of archery targets. J Exp Psychol Hum Percept Perform. 2012 Oct;38(5):1125–1131. doi: 10.1037/a0029036 22731994

[22] Witt JK, Dorsch TE. Kicking to bigger uprights: Field goal kicking performance influences perceived size. Perception. 2009;38(9):1328–1340. doi: 10.1068/p6325 19911630

[23] Jackson K. M. (1979). Fitting of mathematical functions to biomechanical data. IEEE Trans Biomed Eng. 1979 Feb;26(2):122–124. doi: 10.1109/TBME.1979.326551 761932

[24] Winter D. A. (1990). Biomechanics and motor control of human movement (2nd ed.). New York: Wiley.

[25] Hasegawa Y, Fujii K, Miura A, Yamamoto Y. Resolution of low-velocity control in golf putting differentiates professionals from amateurs. J Sports Sci. 2017 Jul;35(13):1239–1246. doi: 10.1080/02640414.2016.1218037 27686139

[26] Hume PA, Keogh J, Reid D. (2005). The role of biomechanics in maximising distance and accuracy of golf shots. Sports Med. 2005;35(5):429–449. doi: 10.2165/00007256-200535050-00005 15896091

[27] Mathers JF, Grealy MA. Motor control strategies and the effects of fatigue on golf putting performance. Front Psychol. 2014 Jan;4:1005. doi: 10.3389/fpsyg.2013.01005 24454298 PMC3888943

[28] van Lier WH., van der Kamp J., and Savelsbergh GJP. Perception and action in golf putting: skill differences reflect calibration. J Sport Exerc Psychol. 2011 Jun;33:349–69. doi: 10.1123/jsep.33.3.349 21659668

[29] Faul F, Erdfelder E, Lang A, Buchner A. G*power 3: A flexible statistical power analysis program for the social, behavioral, and biomedical sciences. Behav Res Methods. 2007 May;39(2):175–191. doi: 10.3758/BF03193146 17695343

[30] Cohen J. Statistical power analysis for the behavioral sciences (2nd ed.). Hillsdale, NJ: Lawrence Erlbaum Associates. 1988.

[31] Wang KP., Yu CL., Shen C., Schack T., Hung TM. A longitudinal study of the effect of visuomotor learning on functional brain connectivity. Psychophysiology. 2023 Dec;30:14510. 38159049 10.1111/psyp.14510

[32] Skibski A., Devorski L., Mangum LC. Providing visual biofeedback using brightness mode ultrasound during a golf swing. J Vis Exp. 2022 Aug;25:186. 36094264 10.3791/64333

